# Investigating the approach of using behavior change techniques in the field of mobile applications: a systematic review

**DOI:** 10.1186/s12913-025-13534-7

**Published:** 2025-10-10

**Authors:** Azam Kheirdoust, Mohammad Reza Mazaheri Habibi, Ali Emadzadeh, Ali Jafarzadeh Esfehani, Fariba Sadat Agha Seyyed Esmaeil Amiri, Kosar Ghaddaripouri, Saeid Eslami

**Affiliations:** 1 Department of Medical Informatics, School of Medicine, Mashhad University of Medical Sciences, Mashhad, Iran; 2 Department of Health Information Technology, Varastegan Institute for Medical Sciences, Mashhad, Iran; 3 Department of Medical Education, School of Medicine, Mashhad University of Medical Sciences, Mashhad, Iran; 4 Metabolic Syndrome Research Centre, Mashhad University of Medical Sciences, Mashhad, Iran; 5 Student Research Committee, Shiraz University of Medical Sciences, Shiraz, Iran; 6 Department of Health Information Management, School of Health Management and Information Sciences, Shiraz University of Medical Sciences, Shiraz, Iran; 7 Pharmaceutical Research Center, Mashhad University of Medical Sciences, Mashhad, Iran

**Keywords:** Behavior change techniques, Mobile application, Patient adherence, Chronic disease

## Abstract

**Background:**

Behavior change techniques (BCTs) are widely used to improve health conditions in individuals. Mobile technologies are increasingly used in health care by facilitating adherence to the recommendations and behavior change. There is inconsistency regarding the usefulness of various BCTs in mobile technologies.

**Objectives:**

This systematic review aimed to assess more effective behavior change techniques in increasing adherence in the management of chronic diseases, promoting behavior change, and improving people’s lifestyles in the field of mobile applications.

**Methods:**

A systematic review of English-language studies was conducted by searching in the reliable scientific databases, including PubMed, Scopus, Web of Science, Embase, and the Cochrane Library. All published articles in the English language that assessed BCTs in behavior change via mobile applications till May 11, 2025, were included in the study. Two techniques were selected as the default techniques in the field of mobile applications based on the behavior change wheel and its mapping to the BCT taxonomy (Michie protocol) and citing scientific reasons.

**Results:**

A total of 8042 articles were obtained, 16 of which were relevant to the objectives of the present study. App Store or Google Play Android applications were evaluated in 10 papers (62.5%), some of which used the Mobile Application Rating Scale (MARS) tool to evaluate applications. Interventions in the majority of the papers (67%) were maintenance treatments and lifestyle modification, including diet and physical activity. BCTs were reported to be effective in all the reviewed studies. The number of BCTs in the studies ranged between one and 53, but the reason for choosing the desired BCTs was not mentioned.

**Conclusions:**

The results of this systematic review show that the use of BCTs in mobile phone applications is important in achieving outcomes. Due to the small body of literature, further studies are necessary to compare the effectiveness of different BCTs in mobile applications.

## Introduction

Chronic diseases are among the leading causes of death and disability worldwide, accounting for more than 70% of annual mortality [1, 2] Their increasing prevalence has placed a significant burden on healthcare systems and economies, particularly in low- and middle-income countries where access to quality services is limited and health inequalities are more pronounced [3–5]. Addressing this global challenge requires scalable and cost-effective solutions that can empower individuals to adopt and maintain healthy behaviors [6, 7].

Mobile health (mHealth) technologies have emerged as a promising approach to address these needs. With more than 2.1 billion smartphone users worldwide and over 100,000 health-related applications available, mobile platforms offer unprecedented opportunities to deliver health interventions at scale [8–10]. These apps can provide self-care education, support lifestyle modifications, and improve treatment adherence across diverse populations [11–13]. However, despite their potential, the extent to which mHealth apps leverage evidence-based strategies remain inconsistent [14–16].

Evidence suggests that many mHealth interventions are developed without sufficient grounding in behavior change theory and fail to systematically integrate behavior change techniques (BCTs) [17–19]. This lack of theoretical underpinning can limit user engagement and reduce long-term effectiveness [20]. The use of BCTs is particularly important because these techniques provide structured approaches to motivate individuals, overcome barriers, and sustain adherence to health behaviors [21, 22].

The Elaboration Likelihood Model (ELM) and the Transtheoretical Model (TTM) are two well-established frameworks that can guide the tailoring of interventions to users’ cognitive engagement and readiness to change [23, 24]. When effectively applied, these models can enable personalized, stage-based interventions that enhance behavioral outcomes [25]. Yet, there is limited systematic evidence examining how these theories are operationalized in mHealth applications and which BCTs yield the most significant impact [26, 27].

User engagement with mHealth tools is also influenced by other factors, including digital literacy, cultural relevance, usability, and personalization of content—dimensions that have been underexplored in the literature [28–30]. Additionally, the absence of standardized reporting on BCT implementation hampers comparisons between studies and prevents the identification of best practices [31, 32].

Similar implementation challenges have also been observed in other digital health interventions such as AI-based systems, where issues like user adoption, digital literacy, and integration into care workflows remain significant [33].

Given these gaps, the present review aims to systematically examine the integration of BCTs in mHealth applications for chronic disease management. Specifically, it seeks to (a) identify the behavioral theories and techniques most frequently used, (b) evaluate their reported effectiveness in supporting adherence to health behaviors, and (c) offer recommendations for designing theory-driven mHealth interventions capable of achieving sustained behavior change.

## Methods

### Study questions

1. What behavior change techniques (BCTs) are most commonly utilized in mobile health (mHealth) applications aimed at modifying health behaviors in patients with chronic diseases?
2. What is the effectiveness of these BCT-based mobile applications in improving self-care, medication adherence, and health-related behaviors among patients with chronic conditions?

### Inclusion and exclusion criteria

Studies were eligible if they:

- Used mobile applications as an intervention tool,
- Employed behavior change techniques (BCTs),
- Targeted any form of health-related behavior change in patients or users,
- Were published in English,
- Reported at least one behavioral or clinical outcome.

Exclusion criteria included:

- Studies lacking sufficient outcome data,
- Studies without full-text availability,
- Non-original research (e.g., reviews, editorials, protocols).

### Information sources and search strategy

A comprehensive search was conducted in the following databases: PubMed, Scopus, Web of Science, Embase, and Cochrane Library. The final search was performed on May 11, 2025.

The search terms included combinations of the following: *“behavior change”*,* “behavior methods”*,* “behavior education”*,* “mobile applications”*,* “software”*,* “mobile health”*,* “mobile app”*,* and “smartphone app”*.

Search strategies were tailored to each database. Mesh terms were used specifically for PubMed to enhance sensitivity. (Table 1)

**Table 1:** Search strategies for databases

Database	Search Strategy
PubMed	(“behavior change“[Mesh] OR “behavior methods” OR “behavior education”) AND (“mobile applications“[Mesh] OR “mobile health” OR “mobile app” OR “smartphone app”)
Scopus	TITLE-ABS-KEY ((“behavior change” OR “behavior methods” OR “behavior education”) AND (“mobile application” OR “mobile health” OR “smartphone app”))
Web of Science	TS= ((“behavior change” OR “behavior methods” OR “behavior education”) AND (“mobile application” OR “mobile health” OR “smartphone app”))
Embase	(‘behavior change’/exp OR ‘behavior methods’ OR ‘behavior education’) AND (‘mobile application’/exp OR ‘mobile health’ OR ‘mobile app’ OR ‘smartphone app’)
Cochrane Library	(“behavior change” OR “behavior modification” OR “behavior education”) AND (“mobile app” OR “mHealth” OR “smartphone application”) *Limited to reviews. *

**Note: ** The search was conducted without time limitations and finalized on May 11, 2025. Search strategies were tailored to each database’s structure. Boolean operators and controlled vocabulary were applied

### Study selection

All identified references were imported into EndNote (version 20), and duplicates were removed. Title and abstract screening were conducted independently by two reviewers. Eligible full texts were then reviewed by both researchers.

Disagreements during selection were resolved through discussion or with the input of a third reviewer. Additionally, the reference lists of all included articles were manually searched to ensure completeness.

### Quality assessment

The quality and risk of bias of the included studies were assessed using the Joanna Briggs Institute (JBI) Critical Appraisal Checklists, selected according to study design:

- Prevalence studies: JBI Checklist for Prevalence Studies.
- Analytical cross-sectional studies: JBI Checklist for Cross-Sectional Studies.
- Quasi-experimental designs: JBI Checklist for Quasi-Experimental Studies.
- Randomized controlled trials: JBI Checklist for RCTs.

Two reviewers conducted the quality assessment independently. Discrepancies were resolved by group discussion.

### Data extraction and synthesis

A standardized form was used for data extraction. The following variables were recorded:

- Study reference.
- Author(s) and year.
- Country.
- Study design.
- Number and type of BCTs.
- Target behavior/disease.
- Reported outcomes.

Due to heterogeneity in study designs and outcome measures, a narrative synthesis was used to analyze the data.

### Theoretical framework

To identify and classify the BCTs used, we employed the Behavior Change Wheel and Michie’s BCT Taxonomy. Based on these frameworks, the Elaboration Likelihood Model (ELM) and the Transtheoretical Model (TTM) emerged as the most commonly applied and effective models in mobile health applications.

**Fig. 1:**
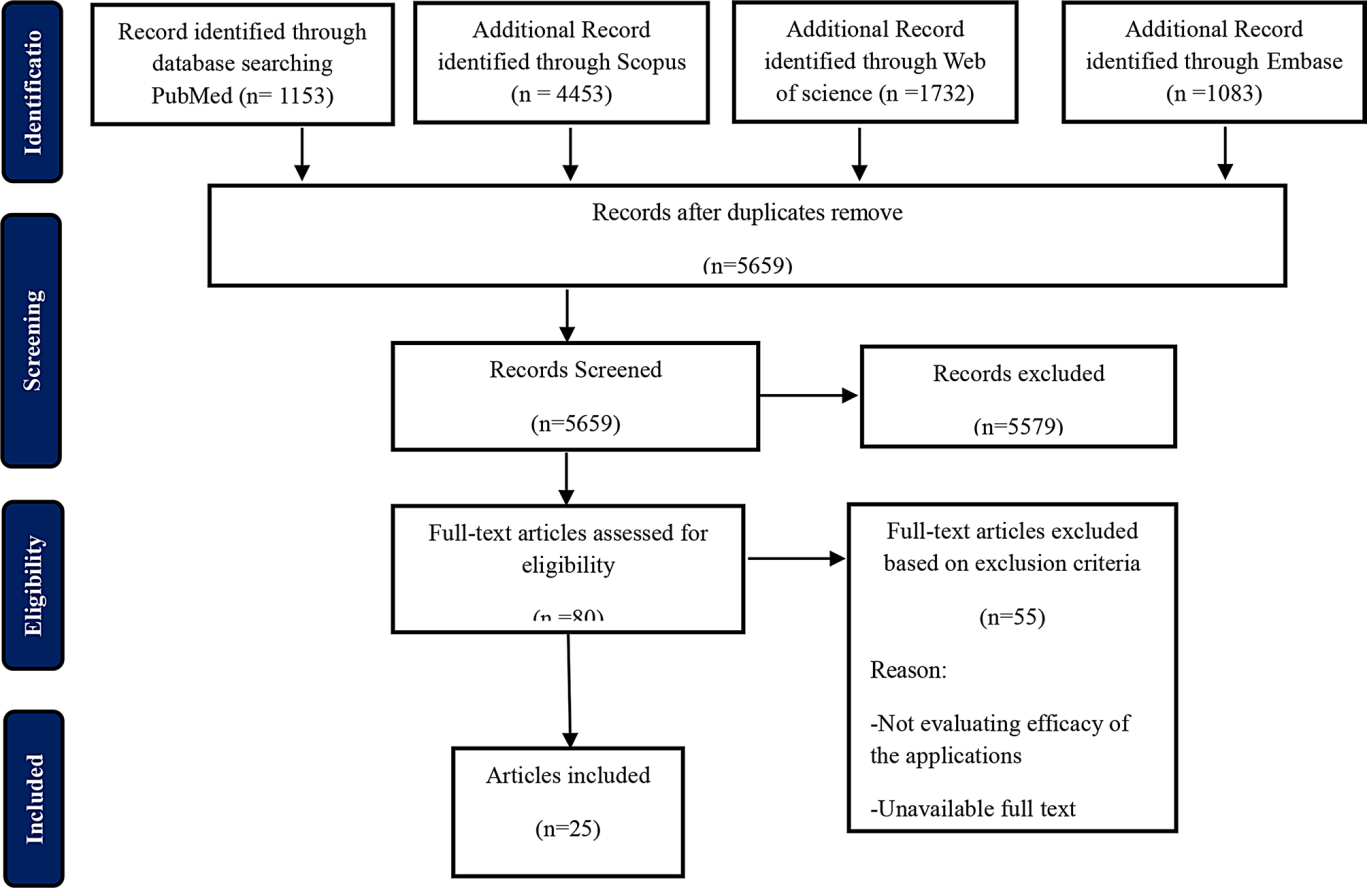
Screening of studies based on the Prisma chart

## Results

Of the primary 8421 papers extracted from searching PubMed, Scopus, Embase, Web of Science databases, and Cochran Library, 80 eligible papers were selected for full-text evaluation based on title and abstract screening. By reviewing full-text papers, 55 papers were excluded and 25 papers were selected for data synthesis. The screening process is summarized in Fig. 1. The characteristics of these studies are reported in Table 2.

Overall, 10 papers (62.5%) investigated Android applications in App Store or Google Play, some of which used the Mobile Application Rating Scale (MARS) tool to evaluate applications. The field of the majority of the papers (67%) included maintenance treatments and lifestyle modification, including diet and physical activity.

All papers pointed to the positive effect of behavior change techniques. The number of BCTs in the studies ranged between one and 53. However, the reason for choosing the desired BCT was not mentioned in none of the papers.

According to the development and testing protocol of linking behavior change techniques to the action mechanism and behavior change wheel in the studies by Michie et al. [18, 22, 32]. Based on the protocol proposed by Michie et al., behavior change wheel is comprised of two layers. The central layer, which included the effective component of motivation in improving each study outcome, and the function layer, which included the functions of education, persuasion, and motivation. Then BCT taxonomy mapping was performed to select two techniques. The identified techniques that were applicable to a wide range of outcomes and populations were Elaboration Likelihood Model (ELM) and Trans Theoretical Model (TTM).

In summary, the reasons for selecting ELM technique were dealing with the mental processing of an individual (information processing) and influencing factors from the outside (decision-making); incorporating persuasion, consideration of the interaction between users and the application, and dividing individuals into two distinct groups [34, 35]. The ELM technique divides individuals into two categories, central and peripheral. Individuals that are identified as central have enough motivation and can read long content and are less prone to losing motivation later. In the contrary, individuals that are identified as peripheral do not have enough motivation and do not like to read long contents and lose their motivation faster compared to individuals who are defined as central [34].

In some studies, the importance of self-monitoring of behavior, behavioral feedback, and the effect of support on insight and behavior in a healthy lifestyle have been mentioned [15, 26, 36]. These features are among the characteristics of the TTM technique. The TTM technique divides the process of behavior change into three stages that include action, maintenance, and relapse. Considering these stages seems necessary in preparing patient educational content in self-management applications.

In summary, the use of two techniques, ELM and TTM, is recommended by default in the field of self-care programs via mobile application for any chronic disease. Other techniques vary according to the type of disease and the component related to it in the behavior change wheel and might not be suitable in majority of behavior change scenarios in all chronic diseases.

**Table 2:** The characteristics of studies

Ref	Author name	Country of study	Year of Study	Study Design	Count of BCT	Type of Disease	Outcome
[12]	De Korte	Netherlands	2018	Search in iTunes and Google Play	**7**	Mental And Physical Health	The presence of BCTs in programs can improve physical and mental health
[25]	Dunn	Canada	2018	iTunes (i.e., for iPhones) and Google Play (i.e., for Androids) Stores	**10**	Physical Activity	should look for a more suitable combination of BCTs, or, were used in low-mobility applications to increase the effectiveness
[11]	Conroy	Pennsylvania	2014	online marketplaces: Apple iTunes (iPhone operating system [iOS]) and Google Play (Android)	**1–13**	Physical Activity	To develop optimized programs, modify healthy lifestyle behaviors, and reduce the burden Non-communicable diseases will be useful
[15]	Kalke	United States	2020	the iOS App Store and the Android Play Store	**30**	Breast Cancer	- Improvement and effectiveness in the general health and well-being of people - The attention and participation of most health experts and scientists of behavioral sciences and communications in the development of the application and adherence to theories in the design of mHealth applications in the continuum of care
[30]	Schoeppe	Australia	2017	iTunes and Google Play stores- MARS	6	Diet, physical activity and sedentary behavior	- Programs with more BCTs are of higher quality - Factors that improve user interaction with the application should be identified. - Designed for specific demographic groups - Be aware of the theories of health behavior change
[29]	Edwards	UK	2016	Apple and Google Play stores	Median number of techniques per app was 14 (range: 5–22)	medical, health and wellness, and health and fitness apps	More research is needed to evaluate the effectiveness of behavior change techniques and evaluate clinical outcomes.
[37]	Davis	United States of America	2019	quasi-experimental - Mobile App Rating Scale (MARS)	Average of 6.6 BCTs (range 3–14	Physical Activity	Positive effect on behavior change - User satisfaction with programs that have more behavior change techniques - More research is needed to understand how these perceptions affect users when choosing an application.
[24]	Ramsey	Ohio	2019	Apple app store and the Google Play Store- MARS	Ranged from 1 to 11 (mean, 4)	Asthma	No studies have documented the most important mHealth BCTs
[38]	Brannon	USA.	2014	iTunes apps	At least one	Physical Activity and Dietary	more cooperation There is a need for pediatric psychologists and technologists to incorporate evidence-based BCTs into the development of appropriate mobile applications.
[27]	Priesterroth	Germany.	2019	Google’s Play Store	Average of 7.4 BCTs (SD = 3.1)	Diabetes	Systematic research on the effectiveness of BCTs and more guidelines for application design are needed.
[26]	DeSmet	Belgium	2019	Cross-Sectional Survey Study	-	24-hour movement behaviors	Preferences were generally the highest for information related to the health consequences of self-monitoring of motor behavior, behavioral feedback, and insight into a healthy lifestyle. - Information about behavioral health Provide results and feedback and social support
[39]	Arrogi	Belgium	2014	Intervention	-	Sedentary Behavior	Positive impact on behavior changes and lifestyle change
[40]	Gilliland	Canada	2015	A quasi-experimental	-	Healthy Dietary Behaviors	Positive impact on sustainable behavior change
[36]	Heneghan	United States	2021	Cross-Sectional Survey	-	Pediatric Acute Lymphoblastic Leukemia	Positive effect on medication adherence - User satisfaction with easy use
[21]	MacPherson	UK	2021	Intervention	28 BCTs	Diabetes	Positive impact on behavior change
[41]	Lorencatto	UK	2013	Before and after	53 BCTs	Smoking Cessation	Positive impact on changing habits and removing unhealthy behavior
[42]	Elena Agachi	Netherlands	2019	quasi-experimental	-	non-communicable diseases	A significant reduction in healthcare costs was observed. In the years 2018 and 2019, there was a decrease of 4.9% and 5.3%, respectively, in healthcare expenses among users of the program. Additionally, there was a notable decrease in costs related to general practitioner services and a reduced need for specialist services. Greater individual engagement with the program (frequency and intensity) was associated with larger cost savings.
[43]	Mona Alhasan	Canada	2024	field study	-	Stress	Improving time management behaviors (such as goal setting, prioritizing, organizing, and tracking progress) Increasing the sense of control over time Reducing the level of stress and anxiety of students Improving self-confidence in the academic field Positive user experience and easy access to the application The effective role of the reward system and social interactions in motivating users As a result, the overall result shows that the intervention program based on the SortOut application was effective in reducing stress, improving time management behaviors, and increasing the sense of control over time.Improving time management behaviors (such as goal setting, prioritizing, organizing, and tracking progress) Increasing the sense of control over time Reducing the level of stress and anxiety of students Improving self-confidence in the academic field Positive user experience and easy access to the application The effective role of the reward system and social interactions in motivating users As a result, the overall result shows that the intervention program based on the SortOut application was effective in reducing stress, improving time management behaviors, and increasing the sense of control over time.
[44]	Dario Baretta	Switzerland	2023	Randomized Controlled Trial, RCT	More than 15	COVID-19	Change in hand hygiene behavior, which includes the number of times hands are properly washed or disinfected in key situations, and change in related psychological variables such as intention, planning, automaticity of behavior, beliefs about consequences and risk.
[45]	Lauren Bell	United Kingdom	2023	micro-randomized trial - MRT	-	Harmful Alcohol Consumption	Increasing user engagement and interaction with the app, reducing alcohol consumption in the short term (i.e. within an hour of receiving the notification), and analyses of the time until users stop using the app (disengagement time) and the effectiveness of notifications on how long users stay in the app.
[46]	Belinda Borrelli	United Kingdom	2024	Pilot Randomized Controlled Trial	More than 10	Smoking and reducing tobacco use	User satisfaction and engagement (management and planning of valuable activities, changes in mood, motivation and confidence in leadership, and reduction in cigarette consumption in the participant group)
[47]	Menna Brown	UK	2023	Participatory Design - co-design		Mental health and lifestyle behaviors in the young population in response to mental health needs after the COVID-19 pandemic	Active participation of young people in the process of designing and producing new content and customizing the site based on their needs and preferences Improving user interaction and activity on the site Increasing mental health and subjective well-being, although statistically significant changes were not observed in mental health assessment scales (such as WEMWBS, PHQ-4, AAQ-II), in the 3-week group, the increase in mental health score was close to significant (P = 0.05) The rate of follow-up and completion of various site activities and modules, and users' comments on the usability and usefulness of the site Adapting the design to the needs mentioned by users and improving their interaction and motivation to continue using the program
[48]	Kayo Waki	Japan	2021	Single-arm pilot study with pre-post evaluation, followed by a post hoc secondary analysis	2	Type 2 Diabetes	86.7% increase in daily steps (from 5436 to 10150 steps per day) Reduction in HbA1c from 8.58% to 7.79% (0.79% decrease) Statistically significant correlations between: Goal completion rate (GC) and increase in steps (r = 0.649, P < 0.001) Coping performance assessment and increase in self-efficacy for control (SI) (r = 0.47, P = 0.004) Goal completion rate (GC) and increase in self-efficacy for specific task (r = 0.446, P = 0.01) Increase in self-regulation (SR) and increase in steps (r = 0.355, P = 0.046) No significant evidence to predict changes in self-efficacy before behavior change in cross-sectional panel modeling
[49]	Kayo Waki	Japan	2021	Randomized controlled trial (RCT) with a 12-month intervention period, comparing an intervention group (using DialBetesPlus mHealth system plus usual care) to a control group (usual care only)		Type 2 Diabetes Mellitus with Diabetic Kidney Disease (DKD), specifically with moderately increased albuminuria (UACR: 30–299 mg/g creatinine)	Primary outcome: 28.8% reduction in UACR (urinary albumin to creatinine ratio) in the intervention group compared to the control group after 12 months (P = 0.029) In ANCOVA analysis adjusting for drugs associated with albuminuria (GLP-1 RA, SGLT-2i, ACEi, ARB), there was a 32.3% reduction in UACR (P = 0.008) 40.3% of patients in the intervention group had a ≥ 30% reduction in UACR compared to 20.3% in the control group (hazard ratio = 1.98, P = 0.019) Secondary outcomes: 0.32% reduction in HbA1c in the intervention group compared to the control group (P = 0.041) Significant improvement in HDL-C (P = 0.041) Non-significant improvement in eGFR (between-group difference: -2.3 mL/min/1.73m², P = 0.141) Significant improvement in BMI in adjusted analyses (P = 0.045) Behavioral outcomes: Significant increase in exercise measurement rate (68.5–81.6%) and daily step count (monthly mean 7552–8693 steps) Significant improvement in self-monitoring of blood glucose (P < 0.001 at 6 and 12 months) No significant improvement in dietary monitoring (food measurement rate decreased from 54.0% to 37.2%) Study retention rate in the intervention group was 93.9% and DialBetesPlus interaction rate was 81.6% at 12 months Exploratory analyses: Multivariate regression showed that change in HbA1c (P = 0.003) and systolic blood pressure (P = 0.010) were associated with change in UACR Change in HbA1c with daily step count (P = 0.045), change in blood glucose (P = 0.002), and BMI (P = 0.011) Proposed mechanism: Intervention improved HbA1c through improved exercise, which in turn reduced UACR Adverse events: No deaths, cardiovascular outcomes, renal endpoints, severe hypoglycemia, or adverse events were reported
[50]	Melina Waranski	Germany	2023	Quasi-experimental controlled trial with a 24-week intervention period, comparing a case manager-assisted eHealth program (RehaPlus+) to a conventional physician-assisted outpatient program (usual care, IRENA). The study was designed as a noninferiority trial with a 6-month follow-up.	3	Coronary Artery Disease (CAD), including patients with ST-elevation myocardial infarction, non-ST-elevation myocardial infarction, stent implantation, or bypass surgery	Primary Results: Physical activity (PA): The RehaPlus + group demonstrated a mean of 182 (SD 208) minutes per week of physical activity at 6 months after discharge from the second phase of cardiac rehabilitation, compared with 119 (SD 175) minutes per week in the usual care group (P = 0.12). The difference between groups was within the prespecified one-sided non-invasiveness margin (half standard deviation: 87.5 minutes), indicating non-invasiveness of RehaPlus + over usual care. Both groups generally demonstrated a significant increase in physical activity from pre-rehabilitation to 6 months later (mean increase of 92 minutes per week, P = 0.001). Analysis of responders showed that 70% (31/44) of the RehaPlus + group were responders compared with 48% (29/61) of the usual care group. Activities of Daily Living (ADLs): The RehaPlus + group showed an ADL level of 443 (SD 538) minutes per week compared with 308 (SD 412) minutes per week in the usual care group at 6 months (P = 0.84). The difference between the groups was at the margin of non-significance, indicating non-significance of RehaPlus+. Both groups showed a significant increase in ADL from pre-rehabilitation to 6 months (P = 0.006). Analysis of responders showed that 46% (20/44) of the RehaPlus + group were responders compared with 33% (20/61) of the usual care group. Secondary outcomes: Physical activity at work and floors climbed per week: Significant increases were observed in both groups (P = 0.03 for work activity, P = 0.02 for floors climbed), but there was no significant difference between the groups (P > 0.05). Psychological well-being (WHO-5 Well-Being Index): Significant increase in both groups (P = 0.001), no significant difference between groups (P > 0.05). Cardiac self-efficacy (CSE): Significant increase in both groups (P = 0.001), no significant difference between groups (P > 0.05). Health-related quality of life (SF-36): Improvement in physical (PCS) and mental (MCS) components in both groups, no significant difference between groups (P > 0.05). Work ability (Work Ability Index): Improvement in both groups, no significant difference between groups (P > 0.05). Other considerations: Loss to follow-up rate: 41% (31/75) in the RehaPlus + group and 27% (22/83) in the usual care group (P = 0.07), mainly due to difficulties in contacting for follow-up. Messages sent: 100% (72 messages) were sent to each patient in the RehaPlus + group as planned. Costs: RehaPlus + cost approximately €540 per patient for 24 weeks compared to €770 for the usual care program (IRENA). Limitations: Self-reported physical activity data, high loss to follow-up rate, and lack of objective measurements.

## Discussion

This systematic review is among the few that comprehensively assessed the effectiveness of behavior change techniques (BCTs) embedded in mobile applications targeting health behavior change across chronic disease populations. While earlier reviews have predominantly focused on identifying BCTs used in mHealth interventions, our review uniquely emphasized their efficacy in promoting behavioral and clinical outcomes.

Overall, the included studies consistently reported positive impacts of BCTs, such as improved self-care, increased adherence to healthy behaviors, and user satisfaction. Among the applied BCT frameworks, the Elaboration Likelihood Model (ELM) and the Transtheoretical Model (TTM) were the most prevalent and effective.

For instance, Davis et al. [37]found that participants preferred mHealth interventions that incorporated a higher number of BCTs, reporting improved usability and perceived effectiveness. Similarly, studies by Arrogi et al. [39] and Gilliland et al. [40] demonstrated sustained behavior change and high engagement levels in interventions with well-structured BCT components.

These findings affirm that both the quality and quantity of applied BCTs significantly influence intervention effectiveness.

However, several studies indicated that although behavior change frameworks (such as the Behavior Change Wheel) are used during intervention design, their integration into app content and delivery remains inconsistent. For example, MacPherson et al. [21] developed 124 BCT-based messages using the BCW but noted that their effect on long-term behavior was unclear. Lorencatto et al. [41] similarly used BCTs for smoking cessation messages but lacked robust outcome data.

A review by Milne-Ives et al. [9], which we compared with our findings, focused mainly on app quality and engagement rather than BCT effectiveness. In contrast, our review adds value by identifying two specific theoretical models (ELM and TTM) that showed superior performance in guiding message tailoring and intervention delivery in chronic disease contexts.

Regarding heterogeneity, the included studies varied in terms of target populations, study design, BCT taxonomy application, and outcome measurement. This variation limited the ability to synthesize results quantitatively (i.e., via meta-analysis), but provided a rich qualitative perspective. Heterogeneity also reflects the diversity of real-world mobile health interventions, underscoring the need for more standardized reporting.

We found that tailoring intervention messages using ELM (central vs. peripheral processing) and TTM (stage-of-change model) can enhance personalization and relevance. For example, individuals categorized under central processing may benefit from longer, in-depth messages delivered less frequently, while peripheral users may prefer short, frequent, and direct messages. The application of TTM allows interventions to align message content with a user’s current stage in the behavior change process.

Strengths of this review include its comprehensive search strategy, inclusion of diverse chronic diseases, and theoretical grounding in both BCT taxonomy and behavioral science. Additionally, our study goes beyond cataloging BCTs by evaluating their practical effectiveness and providing design implications for future interventions.

However, this review also has several limitations. First, we included only English-language studies, which may introduce language bias. Second, due to limited availability of randomized controlled trials (RCTs), we included studies with diverse designs, potentially affecting the robustness of evidence. Third, the lack of detailed outcome reporting in several studies hindered critical comparison across interventions. These factors collectively prevented the performance of a meta-analysis. We recommend that future research includes well-powered RCTs with clinical outcomes, such as laboratory data or anthropometric measures, to strengthen evidence.

In conclusion, our findings suggest that behavior change techniques—particularly ELM and TTM—can significantly enhance the design and effectiveness of mobile health interventions. Despite the growing presence of mobile apps for chronic disease management, the systematic integration of BCTs remains insufficient. Future studies should prioritize rigorous evaluation of BCT-based interventions, with standardized reporting and direct comparisons across frameworks, to advance the evidence base and guide best practices in digital health design.

From a practical standpoint, developers of mobile health applications can leverage the unique characteristics of ELM and TTM to deliver more effective and personalized behavior change interventions. For example, integrating ELM principles allows tailoring message complexity and delivery frequency to user motivation levels, while TTM enables dynamic content delivery based on the user’s current stage of change. Clinicians can also benefit from these frameworks by providing app-based interventions that align with patients’ readiness and engagement levels, ultimately enhancing adherence and health outcomes in real-world settings.

## Conclusion

This systematic review demonstrates the promising role of behavior change techniques (BCTs) in enhancing the effectiveness of mobile health (mHealth) applications for chronic disease management. Among the various BCTs, the Elaboration Likelihood Model (ELM) and the Transtheoretical Model (TTM) were most frequently applied and showed notable effectiveness. ELM’s capacity to tailor persuasive messages based on users’ cognitive processing, and TTM’s structured, stage-based approach to behavior change, contributed significantly to intervention outcomes.

However, evidence remains limited due to a lack of randomized controlled trials (RCTs), heterogeneous study designs, and insufficient reporting of clinical outcomes. Future research should focus on rigorous RCTs with standardized behavioral, clinical, and biometric indicators to validate these techniques.

We also highlight the urgent need for standardized BCT reporting in mobile app studies to enhance replicability, transparency, and real-world implementation of effective digital health interventions.

## Data Availability

No datasets were generated or analysed during the current study.
